# Evaluation of *Trigonella foenum-graecum* L. Plant Food Safety after Lead Exposure: Phytochemical Processes

**DOI:** 10.3390/plants11192526

**Published:** 2022-09-27

**Authors:** Wiem Mnafgui, Valeria Rizzo, Giuseppe Muratore, Hicham Hajlaoui, Amine Elleuch

**Affiliations:** 1 Laboratory of Plant Biotechnology, Faculty of Sciences, University of Sfax, Sfax 3000, Tunisia; 2 Di3A, Dipartimento di Agricoltura, Alimentazione e Ambiente, University of Catania, Via S. Sofia 100, 95123 Catania, Italy; 3 Regional Center for Agricultural Research in Sidi Bouzid, Laboratory of Non-Conventional Water Valuation (INRGREF), University of Carthage, Tunis 9100, Tunisia

**Keywords:** fenugreek, lead uptake, amylase activity, catalase, superoxyde dismutase, flavonoid, phenols

## Abstract

Lead stands as a food contaminant through its accumulation in consumed plants. In this study, the effects of lead (II) chloride (PbCl_2_) and its levels of uptake on morphological and phytochemical responses of fenugreek were assessed to evaluate its tolerance and safety for human consumption. Results revealed that PbCl_2_ (50–2000 mg L^−1^) did not affect the germination rate, but it decreased the radicle length and amylase activity. After three months of Pb treatments, the elemental analysis showed that Pb accumulation was greater in roots than shoots, and it was not present in harvested seeds. The bioaccumulation factor > 1 and the translocation factor << 1 observed for 1000 mg L^−1^ PbCl_2_ suggested appropriateness of fenugreek as a phytostabilizer. Additionally, increased lipid peroxidation, hydrogen peroxide, flavonoid levels and catalase activity were observed in Pb-treated fenugreek. Meanwhile, decreased chlorophyll content was detected under these conditions. In turn, the total phenol was correlated with Pb treatment only in roots. HPLC analysis proved that under Pb stress, gallic acid was the most produced compound in treated roots compared to shoots, followed by quercetin. Syringic and chlorogenic acids were more produced in shoots. In conclusion, fenugreek can be used for Pb phytoremediation and is safe for consumption after Pb treatments in the traditional medicine system.

## 1. Introduction

Plants are exposed to several contaminants present in the environment. Heavy metals (HMs) correspond to a major cause of water and soil pollution in Tunisian industrial and agricultural sites [1]. They can be naturally present in the soil or result from anthropogenic activities such as mining, fossil fuel burning, agriculture, and waste management [2]. In Tunisia, pollution due the increasing use and discharge of lead (Pb) has become a global environmental issue [3], as this element is nonbiodegradable and tends to be absorbed by plants, thus, it accumulates at different levels throughout the food chain, depending on its chemical form, concentration, time of exposure and plant species [4]. Over recent years, Pb contamination of foods has constituted a significant risk to consumers [5]. In response, multiple efforts have been devoted to developing strategies for producing accumulative plants with permissible levels of Pb [6]. They are based not only on manipulating the metal uptake, translocation and immobilization into grain, but also on the use of accumulative plants endowed with adapted mechanisms [7].

Fenugreek (*Trigonella foenum-graecum* L.) is an annual leguminous herb that is extensively cultivated in India and North African countries [8]. In Tunisia, fenugreek is widely cultivated in the northwestern, northern and northeastern regions [9]. It has been investigated not only as a multipurpose medicinal herb through the extraction of phenolic compound to be used in pharmacological field [10], but also in phytoremediation programs [11,12]. In traditional medicine, fenugreek is used to prepare infusions, water and alcohol extracts, etc., to different purposes; notably as an antidepressant, for skin problems, treatment of gastrointestinal disorders and eye diseases. Its applications are various and strictly related to the medical traditions of different countries [13]. Several authors have studied multiple biological actions of fenugreek, for example antibacterial, antidiabetic, hypocholesterolaemic, antioxidative, antihypertensive, hepatoprotective, chemoprotective and immunostimulatory effects [13]. It is indeed a source of saponins, flavonoids, phenols and essential oils [13], which justifies our choice within the framework of the valorisation of this species to be used not only to depollute soils from Pb excess but also own the challenge to produce harvested seeds with admissible levels of Pb.

Previous research has reported that Pb affected the productivity, quality, and secondary metabolites of fenugreek [10]. Its uptake, accumulation and distribution among different plant organs depend on the plant species and concentrations of Pb in soil [6]. Pb toxicity is not only related to the total concentrations in soils, but rather to the mobility of Pb in soil, its bioavailability and its uptake mechanism, which is in turn affected by several factors such as root characteristics, soil physicochemical properties, environmental conditions and plant species [14].

Pb generally enters into the plant system via roots. However, the relationships among different edible plant parts regarding Pb uptake, distribution and accumulation are of great importance. Once Pb enters in excessive doses into the plant, it generates various symptoms of toxicity including reduced germination, stunted growth, chlorosis, necrosis, and reduced shoot and root length, as well as hormonal imbalance and increasing ROS in plants [15]. Phenolic compounds represent a widely diverse group of secondary plant metabolites, serving to protect cells from oxidative stress entailed by free radical species [16]. Several of these compounds play intrinsic physiological and ecological roles being involved in resistance to HM stress [17]. Previous reports demonstrated that the production of different types of polyphenols such as gallic acid, syringic acid and catechin can be stimulated by Pb stress [18].

Therefore, this study aims to evaluate the effects of PbCl_2_ concentration on morphological, oxidant and antioxidant parameters of fenugreek during three months of treatment. This work was achieved through a follow up of Pb accumulation, translocation and immobilization in harvested seeds of fenugreek in order to confirm that only fenugreek seeds exposed to high levels of Pb can be safely consumed. Shoots and particularly roots accumulate some Pb amount. In addition, phenol compounds and flavonoid content were evaluated as Pb-related stress biomarkers.

## 2. Results

### 2.1. Effects of PbCl_2_ Stress on Fenugreek Seed Germination, Radicle Length and Amylase Activity

**Figure 1:**
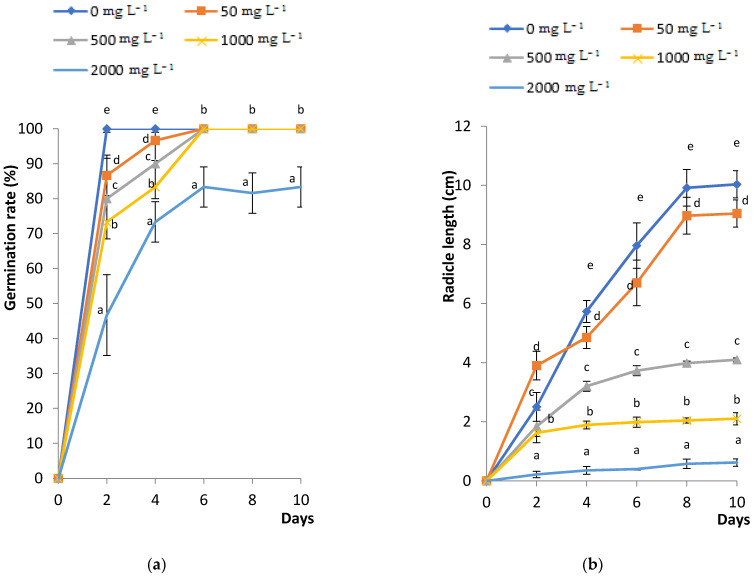
Monitoring of the effects of PbCl_2_ treatment (0, 50, 500, 1000 and 2000 mg L^−1^) on seed germination rate (**a**) and radicle length (**b**) of treated fenugreek seeds over 10 days. All the values are means of three triplicates ± SD of 10 seeds each. ANOVA is significant at *p* < 0.05. Different letters indicate significantly different values at a particular duration (DMRT, *p* < 0.05).

The effects of PbCl_2_ treatments on the germination rate and the radicle length of fenugreek are depicted in Figure 1. The statistical analysis revealed a significant variation (*p* < 0.05) among Pb treatment concentrations, except for 2000 mg L^−1^. By the 6th day of germination, all treated seeds were able to germinate (Figure 1a). Figure 1b demonstrates that increasing Pb concentrations reduced radicle length significantly. In fact, after 10 days of treatment, the radicle length was reduced by 60%, 80% and 95% under 500, 1000 and 2000 mg L^−1^ of PbCl_2_, respectively, compared to the control. The obtained seedlings at the germination stage were puny in the presence of 2000 mg L^−1^ PbCl_2_.

**Figure 2:**
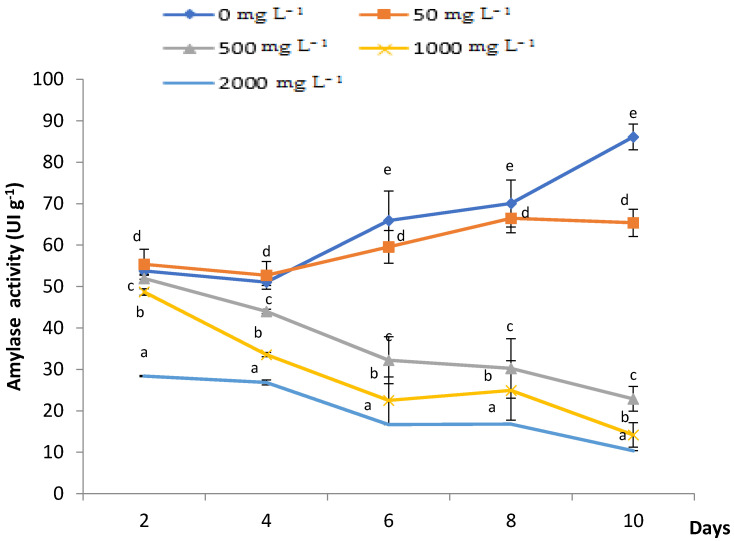
Monitoring of the effects of PbCl_2_ treatment (0, 50, 500, 1000 and 2000 mg L^−1^) on α-amylase activity of treated fenugreek seeds over 10 days. All the values are means of three triplicates ± SD of 10 seeds each. ANOVA is significant at *p* < 0.05. Different letters indicate significantly different values at a particular duration between different treatments (DMRT, *p* < 0.05).

The amylase activity was measured during 10 days of PbCl_2_ treatment at the germination stage (Figure 2). After 2 days of germination in the dark, there were no significant differences recorded in 50, 500 and 1000 mg L^−1^ Pb treatment results, except for 2000 mg L^−1^ where the amylase activity was significantly decreased to 28 UI g^−1^, compared to 56 UI g^−1^ in the control. After 4, 6, 8 and 10 days, the increasing Pb concentrations yielded a significant decrease in the amylase activity, except with 50 mg L^−1^ PbCl_2_ concentration.

### 2.2. Determination of Pb Contents in Fenugreek Tissues

The amount of Pb uptake was measured in roots, shoots and harvested seeds of fenugreek plants after three months of cultivation (Table 1). The results showed that the maximum Pb accumulation was recorded on fenugreek roots treated with 1000 mg L^−1^ PbCl_2_ (0.769 mg g^−1^), and that the minimum metal accumulation was observed on fenugreek shoots treated with 50 mg L^−1^ PbCl_2_ (0.0198 mg g^−1^). Table 1 shows that, with all treatments, the measured Pb concentrations in fenugreek roots were higher than those in shoots. The highest bioaccumulation factor (BAF) of Pb was recorded in fenugreek plants treated with 1000 mg L^−1^ of PbCl_2_ (1.55). The highest translocation factor (TF) was expressed as 0.72 (50 mg L^−1^ PbCl_2_) and the lowest TF as 0.04 (1000 mg L^−1^ of PbCl_2_). The harvested seeds recovered after three months were devoid of lead in both 50 mg L^−1^ and 500 mg L^−1^ PbCl_2_ treatment cases.

### 2.3. Chlorophyll Content

**Figure 3:**
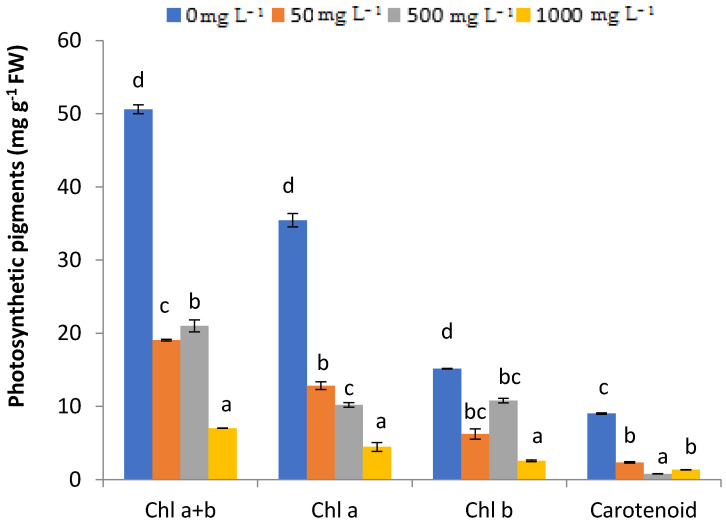
Effects of PbCl_2_ concentrations (0. 50, 500, 1000 mg L^−1^) on chlorophyll content and carotenoid of treated fenugreek after three months of exposure. All the values are mean of triplicates ± SD. ANOVA significant at *p* < 0.05. Different letters indicate significantly different values at a particular chlorophyll pigments and carotenoid (DMRT, *p* < 0.05).

The effect of Pb on the fenugreek plant physiology was assessed through quantifying chlorophyll and carotenoid contents after three months of Pb treatments. The increasing amendment of Pb significantly affected the photosynthetic pigments of fenugreek plants as exhibited in Figure 3. This was clearly inferred while measuring the chlorophyll content, which was inversely proportional to the increasing Pb concentrations. Under 1000 mg L^−1^ concentration, the chlorophyll content was very low (4.46 mg g^−1^ FW) compared to the control, reaching 35.44 mg g^−1^ FW. The statistical analyses were suggestive that Pb stress produced a more pronounced impact when chlorophyll a + b increased. Meanwhile, carotenoid content dropped significantly with the increase in Pb concentration.

### 2.4. Effects of PbCl_2_ Stress on hydrogen peroxide (H_2_O_2_) and lipid peroxide (MalonDiAldehyde, MDA) Levels

**Figure 4:**
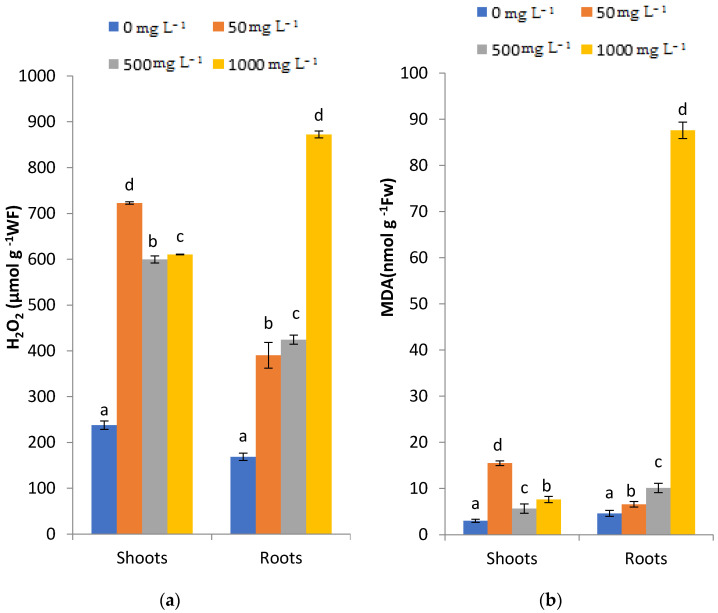
Effects of (0. 50, 500, 1000 mg L^−1^) PbCl_2_ on H_2_O_2_ (**a**) and MDA (**b**) of treated roots and shoots of fenugreek after three months of exposure. All the values are means of triplicates ± SD. ANOVA is significant at *p* < 0.05. Different letters indicate significantly different values in a particular tissue of fenugreek under different treatments (DMRT, *p* < 0.05).

H_2_O_2_ and MDA contents in the shoots and roots of fenugreek seedlings were significantly affected by the introduced Pb doses. The statistical analysis indicated that after three months of treatment, a significant increase in H_2_O_2_ levels occurred under all Pb concentrations compared to the control. H_2_O_2_ contents were more pronounced in shoots compared to roots (Figure 4a). The increasing H_2_O_2_. Content in shoots (Figure 4a) was inversely proportional to the PbCl_2_ concentrations. However, this increase was proportional to the increasing PbCl_2_ concentration in treated roots (Figure 4a). Figure 4b highlights that MDA production was highly significant in roots compared to shoots, and that MDA levels increased in response to increasing concentrations of Pb in fenugreek roots and shoots after three months of treatment. The highest MDA production (88.61 nmol mg^−1^ WF) was observed in seedlings subjected to 1000 mg L^−1^ PbCl_2_.

### 2.5. Effects of PbCl_2_ Stress on Catalase (CAT) and Superoxide Dismutase (SOD)

**Figure 5:**
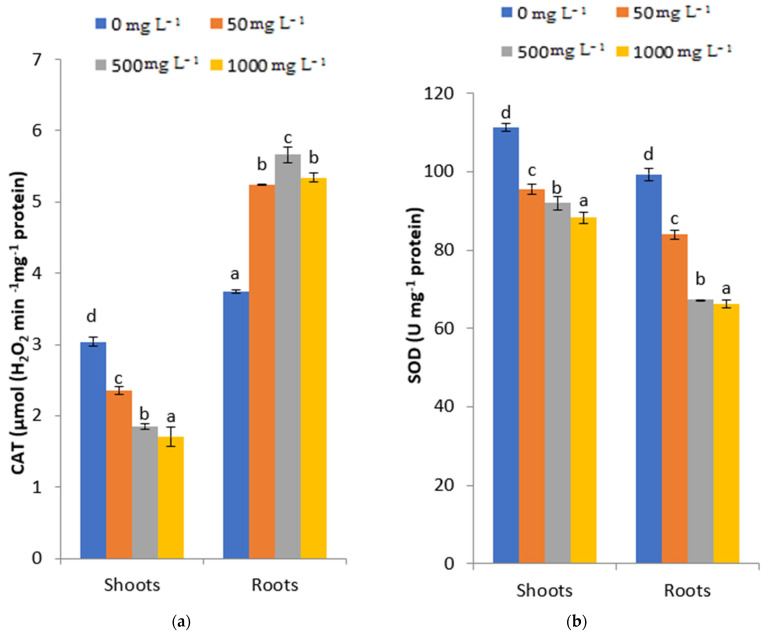
Evaluation of CAT (**a**) and SOD (**b**) in roots and shoots of fenugreek after three months of (0, 50, 500, 1000 mg L^−1^) PbCl_2_ treatments. All the values are mean of triplicates ± SD. ANOVA significant at *p* < 0.05. Different letters indicate significantly different values in a particular tissue of fenugreek under different treatments (DMRT, *p* < 0.05).

The responses of CAT- and SOD-scavenging enzymes to increasing PbCl_2_ concentrations on fenugreek seedlings after three months of treatment are presented in Figure 5a,b. In fenugreek shoots, the statistical analysis showed that the CAT activity decreased significantly with increasing PbCl_2_ concentration, and it was extensively lower than in the control (3.04 µmol H_2_O_2_ min^−1^ mg^−1^ protein). The minimum of CAT activity (1.7 µmol H_2_O_2_ min^−1^ mg^−1^ protein) was recorded under 1000 mgL^−1^. In fenugreek roots, an increased CAT activity was proportional to increasing PbCl_2_ concentration.

The SOD activity decreased gradually after three months of Pb stress compared to the control in both treated shoots and roots. The lowest SOD contents in shoots and roots of fenugreek seedlings treated with 1000 mg L^−1^ of Pb were 88.72 U mg^−1^ protein and 66.18 U mg^−1^ protein, respectively (Figure 5b). The SOD content was more pronounced in Pb-treated roots compared to Pb-treated shoots.

### 2.6. Effects of Lead Stress on Total Phenol and Flavonoid Contents

**Figure 6:**
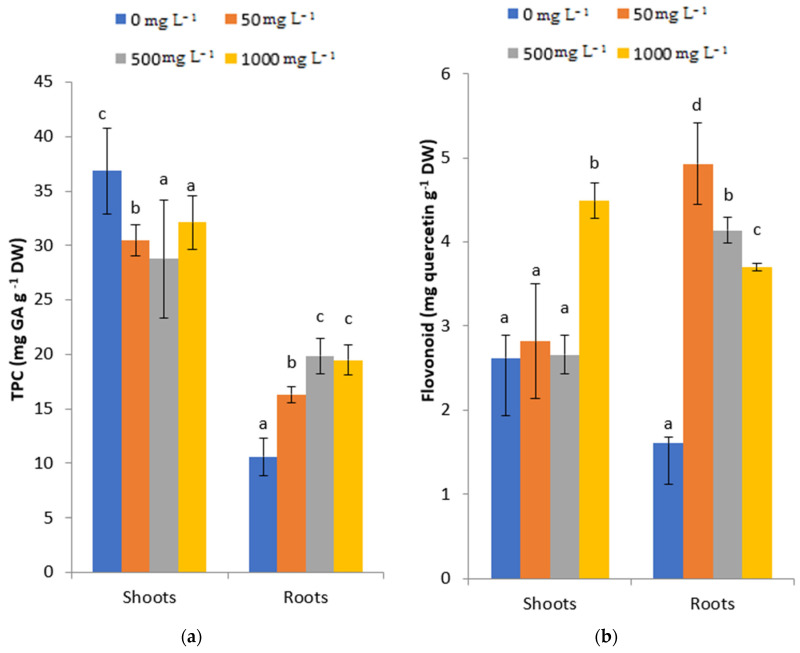
Variation of TPC (**a**) and flavonoid (**b**) contents in roots and shoots of fenugreek after three months of (0, 50, 500, 1000 mg L^−1^) PbCl_2_ treatments. All the values are means of triplicates ± SD. ANOVA is significant at *p* < 0.05. Different letters indicate significantly different values in a particular tissue of fenugreek under different treatments (DMRT, *p* < 0.05).

The data related to total phenol and flavonoid contents are reported in Figure 6a,b. Results revealed that the total phenol content (TPC) was more pronounced in shoots than in roots. Generally, it decreased in fenugreek treated shoots but it enhanced fenugreek treated roots (Figure 6a). The maximum TPC (31.83 mg gallic acid g^−1^ DW) was recorded in control fenugreek shoots.

Figure 6b proved that the flavonoid content in roots was higher than that in shoots under all PbCl_2_ concentrations (50, 500 and 1000 mg L^−1^). Results show that 1000 mg L^−1^ PbCl_2_ stress caused a significant increase in the flavonoid content of fenugreek shoots compared to the other concentrations (50 and 500 mg L^−1^ PbCl_2_) and the control. Yet, results obtained from fenugreek roots indicated a significant increment in the flavonoid content under increasing PbCl_2_ concentrations compared to the control. The highest increase (4.93 mg quercetin g^−1^ DW) was recorded in plants supplemented by 1000 mg L^−1^ PbCl_2_.

### 2.7. Quantification and Identification of Phenolic Compounds

A total of four phenolic compounds were recorded and quantified, as introduced in Table 2. In particular, among phenols with maximum absorbance at 280 nm, the gallic acid was most frequently detected, followed by syringic acid. Table 2 illustrates that the production of gallic acid was more pronounced in roots than in shoots of fenugreek and it was stimulated by the introduced metal compared to the control, while it decreased with increasing concentrations of PbCl_2_. The syringic acid was more frequently quantified in shoots than roots of treated fenugreek. The increasing doses of Pb significantly reduced the production of the syringic acid. Considering compounds with maximum absorbance at 320 nm, the chlorogenic acid was frequently detected in shoots of treated fenugreek, while the quercetin represented 18% of the total amount of phenolic compounds. However, the quercetin was negatively correlated with the increasing PbCl_2_ concentrations.

## 3. Discussion

The plant response to metal toxicity is influenced by its proper developmental stage as well as the type and concentration of the toxicant. The basic objective of this work resides in assessing the effects of Pb on fenugreek seed germination, seedling growth and its levels of accumulation in harvested tissues.

Fenugreek seeds’ germination and radicle length tests revealed that the increase in PbCl_2_ concentrations did not affect the germination rates, except for the 2000 mg L^−1^ of Pb, and decreased the radicle length. These results were in good agreement with those reported by Lamhamdi et al. [19] who emphasized that high Pb concentrations decreased the germination rate of wheat seed germination and reduced growth of seedlings. The decrease in germination rate was explained by Farooqi et al. [20], who reported that the entrance of Pb inside the cell membranes of seeds might delay the germination rate and result in a breakdown of the stored reserves. Other researchers accounted for the effects of copper on decreasing the fenugreek germination rate in terms of seeds permeability, mitosis inhibition, cell wall components’ reduction, Golgi apparatus damage or even polysaccharides’ substitution [21]. On the other hand, we demonstrated that fenugreek seeds were able to germinate even at high doses of PbCl_2_. The above-mentioned result is similar to findings recorded for the *Elsholtzia argyi* plant, where lead was able to accelerate the germination rate and it simultaneously induced a decrease in the roots’ length [22].

Alpha amylase is to one of the most important enzymes used to indicate the progression of germination. Our results revealed that increasing PbCl_2_ concentrations decreased the amylase activity of fenugreek seeds with time. Similarly, Elleuch et al. [21] asserted that the seeds of fenugreek incubated with 20 mM of Cu displayed a progressive decrease in amylase activity over time. Mihoub et al. [23] obtained similar results of α-amylase inhibition under high concentrations of Cd and Cu.

The process of phytoremediation of soils contaminated with HMs depends on two major factors, which are the species of the plant used and the concentration of the introduced or existing metal [24]. It was for essentially this reason that our study focused on the determination of the level of tolerance of fenugreek to Pb stress. From this perspective [24], we first evaluated the response of different plant parts (roots, shoots and seeds) to increasing Pb concentrations (50, 500 and 1000 mg L^−1^). Metal uptake results of our study demonstrated that the maximum Pb accumulation occurred in fenugreek roots rather than shoots. Similar observations were recorded by Xalxo and Keshavkant [10] who clarified that fenugreek treated with 1200 mg L^−1^ of lead was able to accumulate Pb in roots more than shoots. Furthermore, Kaur [12] reported that the Pb accumulation capacity of fenugreek cultivated with 800 mg L^−1^ of Pb during 30 days was more important in roots than shoots. A recent study revealed that fenugreek stressed by zinc (Zn) and aluminum (Al) had an important accumulation factor of these HMs [7]. The phenomenon of metal accumulation in roots more than shoots can be interpreted in terms of the intervention of a chemiosmosis process across the membrane of intact root cells [24]. It could also be correlated with a low TF leading to a higher potential of phytostabilization of HMs in plant roots [12].

After three months of cultivation under Pb stress, harvested fenugreek seeds were recovered and tested. Results showed that they were devoid of Pb except at 1000 mg L^−1^ PbCl_2_ treatment. The level of Pb accumulation remained in the range of allowable doses following the World Health Organization (WHO) recommendation.

Additionally, our plant choice was based on two main factors. The TF, which classifies the plant into an indicator (TF~1), an excluder (TF << 1), or an accumulator plant (TF > 1) and the BAF, which served to the quantification of toxic elements in plants [25]. If the value of BAF is greater than 1.0, it indicates that plants are able to extract metals and therefore they can be selected for phytoremediation of the contaminated soil.

The TF is a measure of the capacity of plants to transfer accumulated metal from the roots to the shoots. Our results highlighted that TF << 1 under the highest Pb concentration (1000 mg L^−1^), which suggests a lack of translocation of Pb from roots to shoots of fenugreek. The obtained BAF exceeded 1 in 500 mg L^−1^ and 1000 mg L^−1^ Pb treatments. This implies that fenugreek roots are a Pb accumulative. Thus, we conclude that fenugreek is considered as Pb phytostabilizer. These findings proved that fenugreek roots are the only tissues that should be collected in phytoremediation attempts. The utility of fenugreek is thus considered, since roots can remain in soil, whereas the upper parts of plant are basically devoid of Pb. The determination of Pb contents in *Sporobolus pyramidalis*, *Cynodon dactylon*, *Imperata cylindrica*, *Eleusine indica*, *Gomphrena celosioides*, *Rhinconspora corymbosa* and *Echinochloa colona* collected from sites contaminated with Pb proved that Pb contents are more pronounced in roots [26]. Hamideh et al. [27] revealed that *Coriandrum sativum* accumulated Pb, especially in roots.

The chlorophyll content is a basic parameter for evaluating plant photosynthetic activity. It can be used as an indicator of pollutant-induced plant stress as well. The photosynthetic indices can be used to determine the average plant tolerance to Pb [22]. The results obtained in this study yielded a significant reduction in chlorophyll a/b content in Pb-stressed fenugreek leaves. In this context, Sofy et al. [17] suggested that the decline of total chlorophyll content in maize treated with Pb was assigned to an increased level of lipid peroxidation. This is supposed to be caused by ROS-mediated chlorosis, which induces chlorophyll degradation and impairment of chlorophyll biosynthesis [28].

The main function of carotenoids is to protect the chlorophyll from a photo-oxidative destruction [4]. Our results indicated that carotenoid contents decreased considerably by Pb stress. Similar results were recorded by Cenkci et al. [29] on *Brassica rapa* plant and by Biteur et al. [30] on *Raphanus sativus,* and proved that Pb stress affected the carotenoid content. However, Zaid [31] emphasized that carotenoid content increased significantly in leaves of menthol plants (*Mentha piperita* L.) treated with cadmium.

The evaluation of H_2_O_2_ and MDA contents in fenugreek roots and shoots exposed to increasing Pb stress was carried out. These two parameters are generally recognized as indicators of the degree of plant cell membrane damage, and they reflect the ROS level under HM stress [32]. This study reported that the fenugreek roots and shoots produced a high content of H_2_O_2_ and MDA in response to increasing concentrations of PbCl_2_. The stimulation of H_2_O_2_ and lipid MDA production can trigger the antioxidant enzymes and activate antioxidant mechanisms [33]. Indeed, some researchers revealed that Pb stress was responsible for the activation of the antioxidative defense system of maize [34].

Alternatively, Shahid et al. [35] stated that the excessive production of H_2_O_2_ can change the redox status of the plant cells and entail the production of polyunsaturated fatty acids to ROS. MDA production was more pronounced in roots compared to shoots of Pb-treated fenugreek. This result was in good accordance with Xalxo and Keshavkant [10], who unveiled that the application of Pb enhanced the production of MDA in roots of treated fenugreek, rather than shoots. Additionally, Kikui et al. [36] and Gopal and Rizvi [37] and Mnafgui et al. [38] demonstrated an increase in lipid peroxidation as a function of Pb concentration in rice and radish, and Fe concentration in fenugreek, respectively.

The evaluation of antioxidant enzymes of fenugreek treated with increasing Pb concentrations (0, 50, 500 and 1000 mg L^−1^) displayed a slight decrease in SOD in the roots and shoots of fenugreek. Basically, superoxide is the first free radical to be produced during HM stress and it is considered to be the first barrier to ROS. SOD quickly converts superoxide radicals into hydrogen peroxide and oxygen [39]. The reduction in SOD in our work may be assigned to Pb stress damaging the antioxidant enzymatic systems [40]. Similar results were recorded with other plants subjected to heavy-metal stress such as *Coriandrum sativum* L. treated with Pb [27].

The activities of CAT in fenugreek shoots treated with 50 and 500 mg L^−1^ of PbCl_2_ were extensively lower than the control. CAT belongs to the antioxidant system of plants, and it controls the ROS levels by inhibiting the overproduction of H_2_O_2_ in their cells. Thus, the decline of CAT activity could be interpreted in terms of the possible delay in H_2_O_2_ removal, the inactivation of enzyme protein because of ROS and the existence of toxic peroxides mediated by CAT or by severe oxidative damages imposed by Pb stress [41].

Plants produce phenolic compounds in response to environmental stress, including HMs contamination, to protect themselves from oxidative damages. Our results showed that the total phenol content increased in roots compared to shoots of fenugreek treated seedlings. However, Pb-induced stress enhanced the production of flavonoids in shoots compared to roots. In both cases, the increased Pb concentrations caused a reduction in the phenol and flavonoid levels. The accumulation of phenol and flavonoid in response to lead was reported by Zulfiqar et al. [40]. They suggested that this accumulation could be related to active scavenging radicals.

Exposure to HMs increases the production of a wide range of phenolic compounds in plants. They exhibit an important potential of chelating metals and have been considered as electron-donating agents. In this study, the HPLC results suggested that the major phenolic compounds produced under PbCl_2_ stress are gallic acid, followed by syringic acid and quercetin, respectively. The production of gallic acid was positively correlated with the total phenols compound, while the two other compounds were negatively correlated. The present research demonstrated that most of the samples showed a correlation between the phenol and flavonoid contents, antioxidant activity and Pb concentrations in plant shoots and roots. This confirmed that the Pb stress level and production of phenolic compounds with antioxidant activity were directly connected. Similar results were obtained by Sevindik et al. [42], who revealed that phytochemical analysis of *Leucoagaricus leucothites* (Vittad.) under Pb stress proved the presence of important phenolics such as gallic acid. Kisa et al. [43] illustrated that tomatoes cultivated under increasing Pb concentrations had decreased production of benzoic acid. *Imperata cylindrica* treated with high levels of Cu increased the levels of phenolic acids in shoots [44].

## 4. Materials and Methods

### 4.1. Seed Germination, Radicle Length Measurements and α-Amylase Activity Assay

Commercial seeds of *Trigonella foenum-graecum* L. were surface-sterilized and germinated according to Elleuch et al. [21]. The seed germination test was performed on Petri dishes containing a Whatman filter paper moistened with 10 mL of increasing concentrations of PbCl_2_ solutions (50, 500, 1000 and 2000 mg L^−1^). Control seeds were those obtained without the addition of PbCl_2_ (0 mg L^−1^). Germination started from the moment of radicle protrusion to the seed coat. The seeds germination rate was assessed every 2 days (Germination Rate (GR) = (n/N) × 100)), where n is the number of germinated seeds and N is the number of total seeds in each Petri dish. The radicle length was measured in centimeters (cm) every 2 days, by starting from the radicle-seed junction to the tip of the longest root. Lastly, the α-amylase was assayed according to the 3,5 dinitrosalicylic acid method [11].

### 4.2. Plant Growth Conditions

Ten seeds of fenugreek were planted in plastic pots, each containing 1 Kg of inert peat substrate and grown in a glasshouse with an adjustable thermostat (temperature 25 °C and photoperiod 16 h) in the presence of PbCl_2_ mixed in soil. Beyond the objectives of the evaluation of germination rate, radical length and amylase activity, these experiments allowed us to select 0, 50, 500 and 1000 mg L^−1^ as the best PbCl_2_ concentrations in all further experiments. Each planted pot was watered manually with 100 mL of distilled water every couple of days to avoid the filtration of water.

### 4.3. Pb content, Translocation Factor and Bioaccumulation Factor Determination

Shoots, roots and seeds harvested after three months of PbCl_2_ treatments were dried at 60 °C for 72 h, powdered and then digested with a mixture of acid solution containing HNO_3_ and HClO_4_ (2/1 ratio). Obtained extracts were evaporated at 180 °C until a colorless solution was obtained. The final volume of 25 mL with the addition of distilled water was used in atomic absorption spectrometry (Perken Elmer model 2380) analysis in order to determine the Pb content according to Malik et al. [45].

Data of TF, and BAF were calculated following Malik et al. [45] protocol with:

TF = Pb concentration in shoots/ Pb concentration in roots;

BAF = Pb concentration in roots/ Pb concentration in soil.

### 4.4. Leaf Chlorophyll Content

Chlorophyll content measurement was performed as described by Akinci et al. [46]. Approximately, 1 g of fresh leaves was homogenized with a mortar and pestle in 1 mL of acetone (80 %) in the dark.

### 4.5. H_2_O_2_ and MDA Determination

The total hydrogen peroxide (H_2_O_2_) content was extracted and assayed as described by Sagisaka [47] in the roots and shoots of fenugreek seedlings under a concentration of 0, 50, 500 or 1000 mg L^−1^ PbCl_2_. Fresh samples were ground thoroughly with a pestle and mortar in TCA 0.1% (*v/v*) and centrifuged at 12000 rpm for 15 min. The reaction mixture consisted of potassium phosphate (0.1 M, pH 7), potassium iodide (1M) and crude supernatant extract. The absorbance was measured at 390 nm.

The level of lipid peroxides was calculated as MalonDiAldehyde content, following the method demonstrated by Duan et al. [48]. Freshly harvested root and shoot samples (0.5 g) were mixed with 5 mL of 1% (*v/v*) trichloroacetic acid (TCA). The homogenized mixture was centrifuged at 10,000 rpm for 10 min. The reaction medium contained the supernatant and 1 mL of 0.5% (*w/v*) TBA (in 20% TCA). The mixture was incubated at 90 °C for 30 min and then was immediately cooled in an ice bath. After centrifugation at 10000 rpm for 10 min, the optical density (OD) was measured at 532 nm.

### 4.6. CAT and SOD Determination

Root and shoot samples of the fenugreek plants were prepared for CAT and SOD analysis. The mixture was prepared by homogenizing 0.1 g of frozen tissue with a mortar and pestle in a 1mL potassium phosphate buffer (0.1 M, pH 7) in order to extract soluble proteins. The tissue homogenate was then centrifuged at 13000 rpm for 15 min at 4 °C [21].

Referring to Aebi [49], the CAT activity was achieved by monitoring the disappearance of H_2_O_2_ for 10 min at 240 nm. The CAT activity was determined using the extinction coefficient (39 mM^−1^ cm^−1^) and expressed in terms of µmole H_2_O_2_ min^−1^ mg^−1^ protein.

Total SOD activity rests upon measuring its aptitude to inhibit the photochemical reduction of nitroblue tetrazolium chloride (NBT). This experiment was carried out following the Beyer and Fridovich [50] protocol. The reaction solution contained 50 mM phosphate buffer (pH 7.8), 0.1 mM EDTA, 13 mM methionine, 75 mM NBT, 2 mM riboflavin and 25 µL enzyme extracts. Tubes were then shaken and kept under a light source (20 W fluorescent tubes) for 15 min. Next, they were placed in the dark for 10 min. Absorbance was determined at 560 nm.

### 4.7. Determination of the Total Phenolic and Flavonoid Content

The methanolic extraction was conducted relying on the methods of Salehi et al. [51]. The TPC of the extract was determined using the Folin–Ciocalteu (FC) assay, as highlighted by Sevindik et al. [42]. The absorbance was determined at 765 nm, and a calibration curve was prepared using standard solution of gallic acid. Findings were indicated as mg gallic acid g^−1^ of dry weight (DW).

The flavonoid content was estimated according to the Hanafy and Akladious [52] protocol. The absorbance was determined at 430 nm using quercetin as a standard. Total flavonoids were indicated in terms of quercetin g^−1^ DW.

### 4.8. Determination of Phenols by HPLC

Samples were prepared as reported by Akrimi et al. [53]. Notably, each extract (2 mL) was filtered with a syringe PTFE filter (0.45 μm) (Albet, Barcelona, Murcia, Spain) and injected into the HPLC system (Thermo Finnigan Surveyor, Waltham, MA, USA), equipped with a UV–VIS detector. The column was a *Kinetex*^®^ C18 EVO (250 *×* 4.6 mm, 5 µm) (Phenomenex, Torrance, CA, USA), fitted with a guard cartridge packed with the same stationary phase. Solvents and HPLC programs were those described by Rizzo et al. [54]. Standards used in the experiment were gallic, syringic and chlorogenic acid in addition to quercetin. They were injected at different concentrations from 10 to 1000 mg kg^−1^ to obtain suitable calibration curves.

Chromatograms were recorded at 280 and 320 nm; all samples were assayed in triplicate.

### 4.9. Statistical Analysis

All experiments were carried out in a randomized block design. Statistical analysis was conducted using SPSS version 20.0. Data were expressed as mean ± standard deviation of three replicates. One-way analysis of variance (ANOVA) was implemented to all data in order to corroborate their variability and guarantee the goodness of fit of results. Duncan’s multiple range test (DMRT) was employed to emphasize the important differences between repeated experiments and the means, which were compared applying the least significant difference (Duncan’s multiple range) test; *p* < 0.05 was assumed to indicate a statistically significant difference.

## 5. Conclusions

This work proved the outstanding capacities of fenugreek plants to tolerate and remove Pb metal stress under increasing concentrations, reaching up to 2000 mg L^−1^ in the germination test and 1000 mg L^−1^ in the test of fenugreek seeds planted in pots. Results also confirmed that levels of Pb accumulation in harvested seeds and other tissues of fenugreek were in permissible levels. Indeed, this urges the use of fenugreek as its manufacturing necessitates short-term and low-cost phytoremediation strategies. Increased H_2_O_2_ and MDA levels reflected an oxidative response of fenugreek under Pbcl_2_ > 50 mg L^−1^. The increasing activity of CAT, flavonoid and phenol contents allowed to fenugreek plants to overcome oxidative Pb supply. In addition, PbCl_2_ > 500 mg L^−1^ enhanced the production of special types of phenols (gallic acid and quercetin) in roots of treated fenugreek. This work paves the way for fruitful and constructive applications in the pharmaceutical field, as harvested plant tissues (roots and shoots) and harvested seeds can be invested safely in the food pharmaceutical sector and still be attractive towards consumers that appreciate the importance and use of traditional medicine systems.

## Figures and Tables

**Table 1:** Pb content in harvested roots, shoots and seeds of stressed fenugreek seedlings under PbCl_2_ treatment (0, 50, 500 and 1000 mg L^−1^) and evaluation of TF and BAF. All the values are means of triplicates ± SD. ANOVA is significant at *p* < 0.05. Different letters indicate significantly different values at particular lead concentrations (DMRT, *p* < 0.05).

PbCl_2_ Concentration (mg L^−1^)	Content of Pb in Shoots (mg g^−1^ DW)	Content of Pb in Roots (mg g^−1^ DW)	Content of Pb in Seeds (mg g^−1^ DW)	Translocation Factor (TF)	Bioaccumulation Factor (BAF)
0	0a	0.0009 ± 0.002 a	0	0	0
50	0.0198 ± 0.003 b	0.0275 ± 0.008 c	0	0.72	0.44
500	0.0281 ± 0.006 c	0.255 ± 0.005 b	0	0.11	1.32
1000	0.031 ± 0.005 c	0.769 ± 0.031 d	0.0005 ± 0.0001	0.04	1.55

**Table 2:** Effects of PbCl_2_ treatment (0, 50, 500 and 1000 mg L^−1^) on phenolic compound concentrations. All the values are means of triplicates ± SD. ANOVA is significant at *p* < 0.05. Different letters indicate significantly different values at particular lead concentrations (DMRT, *p* < 0.05).

PbCl_2_ Concentration (mg L^−1^)	Gallic Acid (mg kg^−1^)	Syringic Acid (mg kg^−1^)	Chlorogenic Acid (mg kg^−1^)	Quercetin (mg kg^−1^)
**Shoots**				
0	0.105 ±0.014 ^c^	0.040 ± 0.01 ^c^	n.d.	n.d.
50	0.104 ± 0.009 ^c^	0.027 ± 0.039 ^b^	n.d.	0.004 ± 9 × 10^−5 b^
500	0.095 ± 0.008 ^b^	0.026 ± 0.034 ^b^	0.005 ± 0.002 ^b^	0.002 ± 6 × 10^−7 a^
1000	0.078 ± 0.001 ^a^	0.022 ± 0.001 ^a^	0.003 ± 0.0012 ^a^	n.d.
**Roots**				
0	0.105 ± 10^−4 a^	0.107 ± 0.004 ^a^	0.002 ^a^	0.0034 ± 8 × 10^−8 b^
50	0.198 ± 2 × 10^−4 d^	n.d.	n.d.	0.0066 ± 3 × 10^−4 d^
500	0.132 ± 5 × 10^−3 c^	n.d.	n.d.	0.0044 ± 1 × 10^−3 c^
1000	0.103 ± 9 × 10^−3 b^	n.d.	n.d.	0.0031 ± 14 × 10^−5 a^

n.d = not detectable.

## Data Availability

Data sharing is not applicable as new data were generated or analyzed during this study.
